# Modelling an alternative lipophilicity scale of bisphenols using biomimetic chromatography: Relevance to membrane-driven baseline toxicity

**DOI:** 10.5599/admet.3169

**Published:** 2026-03-19

**Authors:** Krzesimir Ciura, Julia Nicman, Szymon Zdybel, Giacomo Russo, Lucia Grumetto, Katarzyna Ewa Greber, Anita Sosnowska, Joanna Dołżonek, Karolina Jagiello

**Affiliations:** 1 Department of Physical Chemistry, Faculty of Pharmacy, Medical University of Gdańsk, Al. Gen. J. Hallera 107, 80-416, Gdańsk, Poland; 2 Laboratory of Environmental Chemoinformatics, Faculty of Chemistry, University of Gdansk, Wita Stwosza 63, 80-308, Gdansk, Poland; 3 QSAR Lab Ltd., Trzy Lipy 3 St., 80-172 Gdańsk, Poland; 4 School of Applied Sciences, Sighthill Campus, Edinburgh Napier University, 9 Sighthill Ct, EH11 4BN Edinburgh, United Kingdom; 5 Department of Pharmacy, School of Medicine and Surgery, University of Naples Federico II, Via D. Montesano, 49, 80131, Naples, Italy; 6 Department of Environmental Analysis, Faculty of Chemistry, University of Gdansk, Wita Stwosza 63, 80-308, Gdansk, Poland

**Keywords:** Immobilized artificial membrane, phospholipid affinity, endocrine disruptors, baseline toxicity, sphingomyelin column

## Abstract

**Background and purpose:**

Bisphenol A and its structural analogues are ubiquitous environmental contaminants and potential endocrine disruptors, creating a need for mechanistically relevant descriptors that support early hazard assessment. This study asked whether lipophilicity indices derived from biomimetic chromatography better reflect baseline toxicity and membrane-relevant behaviour of bisphenols than commonly used in silico log *P* / log *D* descriptors.

**Experimental approach:**

A set of 18 bisphenol derivatives was analysed using phosphatidylcholine- and sphingomyelin-functionalised stationary phases and a conventional C18 column to obtain chromatographic hydrophobicity indices, which were then compared with predicted log *P* / log *D* values and related to mechanistically diverse toxicity endpoints (in vitro cytotoxicity in mammalian cells, ex vivo cardiotoxicity assessed as vasodilation, and aquatic toxicity towards Daphnids).

**Key results:**

Biomimetic chromatographic indices showed consistently stronger and more coherent relationships with biological activity than theoretical descriptors. They also uniquely captured structural effects, such as positional isomerism, which were largely indistinguishable using theoretical lipophilicity indices.

**Conclusion:**

These findings support biomimetic chromatography as an early-tier IATA tool, providing membrane-relevant experimental indices linking bisphenol lipophilicity with baseline toxicity.

## Introduction

The release of hazardous chemicals into the environment occurs through diverse human-induced processes, potentially leading to adverse effects on human health and ecosystems. Among these contaminants, bisphenol A (BPA) stands out as one of the most pervasive environmental [1]. Its extensive application, along with its analogues, spans the manufacture of various polycarbonates and epoxy resins, which are commonly employed in the manufacture of food-contact materials, can linings, bottle cap production, and certain dental sealant components. As a known endocrine disruptor, BPA exhibits both estrogen-mimicking and anti-androgenic activities, leading to adverse effects across various tissues and organs [2]. The reproductive, immune, and neuroendocrine systems are particularly susceptible to BPA-induced damage [3]. Furthermore, recent animal studies have revealed BPA's capacity to promote carcinogenesis and mutagenesis [4]. Reflecting growing regulatory concern, the European Commission decided in 2019 to restrict the use of BPA in materials intended to come into contact with food, following a risk assessment conducted by the European Food Safety Authority (EFSA). By applying the precautionary approach and in response to EFSA’s 2023 re-evaluation, which significantly lowered the tolerable daily intake for BPA, the Commission adopted Regulation (EU) 2024/3190 in December 2024. This new regulation bans the use of BPA and other bisphenols with similar hazardous properties in all food-contact materials, marking a comprehensive step toward minimizing human exposure to these endocrine-disrupting chemicals.

In response to these restrictions, numerous bisphenol analogues have been produced as potentially safer alternatives to BPA, offering comparable functional performances [5]. Despite the availability of these substitutes in the market and their entry into the environment, the safety of BPA analogues still remains controversial [6]. However, their toxicological effects have not been thoroughly investigated or confirmed [5].

Historically, assessment of chemical safety has predominantly depended on animal testing. Although this traditional paradigm has yielded valuable toxicological information, it faces growing criticism owing to ethical concerns, high costs, and its restriction of the number of compounds that can be tested *in vivo*. As a result, only a small fraction of the vast number of industrial and environmental chemicals are ever evaluated for their potential biological effects. In response to these challenges, the past few decades have seen the rise of New Approach Methodologies (NAMs), including *in vitro* assays, *in silico* modelling, and advanced omics technologies [7,8]. These innovative approaches aim to provide mechanistic insights into toxicity pathways and offer more human-relevant, efficient, and ethical alternatives to conventional *in vivo* studies.

Although NAMs-based scientific promise for hazard assessment, their regulatory adoption remains challenging. Their diverse data types and varying levels of biological and toxicological relevance necessitate structured interpretation frameworks to ensure consistency, reliability, and transparency in safety assessments. This requirement led to the creation of Integrated Approaches to Testing and Assessment (IATA), a framework proposed by the OECD to integrate various approaches into cohesive, weight-of-evidence-based decision-making strategies [9].

Since the groundbreaking publication of Meyer [10], lipophilicity has been recognized as one of the most important factors determining the toxicity of chemical substances. Moreover, highly lipophilic compounds preferentially partition into lipid-rich tissues, leading to bioaccumulation and potential biomagnification across trophic levels, even at low environmental concentrations. The partition coefficient between *n*-octanol and water (log *P*), introduced by Hansch in the 1960s [11,12], has long been the standard measure of lipophilicity. However, recent advances in the field have yielded more biorelevant techniques that enable more precise evaluation of physiological distribution and bioaccumulation potential.

Biomimetic chromatography roots in the pharmaceutical sector, where Valko and her team introduced the concept of immobilized artificial membrane chromatography (IAM), proposed by Pidgeon [13] as a technique to assess potential drug candidates [14,15]. Biomimetic chromatography offers numerous benefits, including rapid analysis, minimal sample requirements, high reproducibility, and durability, as these experiments use high-performance liquid chromatography (HPLC) [14,16]. These advantages contributed to the popularity of biomimetic chromatography in both industry and academia [17-20]. Biomimetic chromatography has recently been utilized to analyze substances of toxicological interest, demonstrating a strong correlation between chromatographic results and both cellular and *in vivo* toxicity [6,21]. Some research has shown that biomimetic chromatography can be used to estimate aquatic toxicity [17,22-24], bioconcentration [25], and ADME properties [26] of toxicologically relevant substances. Nonetheless, the application of biomimetic chromatography in toxicology remains in its early stages.

This study examines the application of IAM chromatography as a potential alternative method for assessing the lipophilicity of bisphenols. To offer novel insights into the interactions between phospholipids and bisphenols, a library of 18 bisphenol analogues was analysed using two complementary stationary phases: a commercially available IAM column coated with phosphatidylcholine and an in-house developed sphingomyelin-bonded column. Subsequently, the biochromatographic data were correlated with selected toxicity endpoints, including cytotoxicity, cardiotoxicity, and aquatic toxicity, thereby underscoring the broader applicability of this approach for predicting bisphenol behaviour and risk.

Importantly, this study also highlights the relevance of biomimetic chromatography within the framework of NAMs-anchored IATAs. By providing quantitative, human-relevant, and non-animal data on compound-membrane interactions, IAM chromatography can contribute to the mechanistic understanding of chemical toxicity pathways and support the integration of physicochemical and biological information in IATA-based decision-making. Thus, this work demonstrates how biomimetic chromatography can complement existing *in vitro* and *in silico* methods, advancing the replacement, reduction, and refinement (3Rs) of animal testing in chemical safety assessment.

## Experimental

### Chemical reagents

LC-grade dimethyl sulfoxide (DMSO) and 2-propanol, both suitable for HPLC, were from Sigma-Aldrich (Steinheim, Germany), whereas HPLC-grade acetonitrile (ACN) was purchased from Chempur (Piekary Śląskie, Poland). Ammonium acetate for buffer preparation was purchased from POCH (Gliwice, Poland). Water was purified by using the Millipore Direct-Q 3 UV Water Purification System (Millipore Corporation, Bedford, MA, USA). The reference substances used to calibrate the C_18_ and IAM columns were purchased as follows: acetanilide, butyrophenone, and octanophenone (Alfa Aesar, Haverhill, USA); acetophenone, benzimidazole, colchicine, indole, paracetamol and theophylline (Sigma-Aldrich, Steinheim, Germany); heptanophenone, hexanophenone, propiophenone and valerophenone (Acros Organic, Pittsburg, USA). All substances utilised were of HPLC-grade purity.

### Analytes

Bisphenol A (BPA), bisphenol B (BPB), bisphenol C (BPC), bisphenol E (BPE), bisphenol G (BPG), bisphenol S (BPS), bisphenol BP (BPBP), and 2,2-bis(4-hydroxyphenyl)-4-methylpentane (BPS-MAE) were purchased from LGC Standards (Luckenwalde, Germany); 4,4'-bisphenol F (4,4'-BPF), 2,2'-bisphenol F (2,2'-BPF), 2,4'-bisphenol F (2,4'-BPF) and bisphenol P (BPP) were purchased from Alfa Chemistry (New York, USA); bisphenol Z (BPZ), bisphenol AP (BPAP), bisphenol AF (BPAF), bisphenol PH (BPPH), bisphenol FL (BPFL), and bisphenol A diglycidyl ether (BADGE) were purchased from Sigma-Aldrich (Missouri, USA). The 2D structures and CAS numbers of the investigated bisphenols are summarised in Table S1 in the Supplementary material. Prior to conducting HPLC analyses, all compounds under study were dissolved in DMSO to a concentration of 1 mg mL^-1^ and then stored at 2 to 8 °C. These stock solutions were subsequently diluted to 50 μg mL^-1^ and used in HPLC experiments. Working solutions were freshly prepared on the day of analysis, filtered through a 0.45 μm syringe filter (Chromafil XTRA PET 45/25 GmbH & Co., Germany), and immediately used for chromatographic measurements.

### Biomimetic chromatography analysis

IAM-HPLC and C_18_-HPLC measurements were performed using the protocols developed by Valko and co-workers [15,27] and implemented in our laboratory [28-30]. These methods provided determination of lipophilicity and phospholipid affinity, as represented by chromatographic hydrophobicity index (CHI) values, through a gradient elution experiment and comparison with standard substances. Each chromatographic experiment was conducted using the Prominence-1 LC-2030C 3D HPLC system, managed by LabSolution software. The study employed chromatographic columns with different chemical modifications of their stationary phases, including IAM.PC.DD2 (10×4.6 mm x 10.0 μm; Regis Technologies; USA) and C18 Hypersil GOLDTM (50×4.6 mm; 5.0 μm; Thermo Scientific, USA). All columns were paired with guard columns featuring the same stationary phases as the main column. For IAM-HPLC and C18-HPLC analyses, mobile phase A consisted of a water solution of 50 mM ammonium acetate (VWR International, Leuven, Belgium), adjusted to physiological pH 7.4 using concentrated ammonia solution (Avantor Performance Materials Poland S.A., Gliwice, Poland). Ultrapure water for mobile phase preparation was sourced from a Milli-Q water purification system (Merck Millipore, Darmstadt, Germany) with a resistivity of 18.2 MΩ.

For the measurement of phospholipid binding, the mobile phase B consisted of ACN, employing a linear gradient from 0 to 85 % B over 5.25 minutes, followed by a maintenance at 85 % ACN for 0.5 minutes. The flow rate of the mobile phase was set at 1.5 mL min^-1^, and the IAM.PC.DD2 column was maintained at a temperature of 30 °C. The *R*^2^ coefficient for the calibration mixture was 0.999.

For measuring lipophilicity, the solvents and flow rate matched those used in IAM chromatography. The C18 Hypersil GOLDTM column was kept at 40°C. Likewise, a linear gradient was employed from 0 to 5.25 minutes, but in this instance, it ranged from 2 to 98 % ACN, with the highest concentration of phase B held for 2.5 minutes. The *R*^2^ coefficient for the calibration mixture was 0.976.

Detection was conducted in the UV region, with wavelengths ranging from 190 to 300 nm. A 5.0 μL volume was injected, and each compound was analysed in triplicate, with retention time differences never exceeding 3 %.

IAM.SPH experiments were conducted using an in-house fabricated IAM.SPH analytical column (10 μm, 100×2.1 mm, 30.0 nm pore size) [31], generously gifted by the Separation Science Group of Ghent University. A total of 0.821 mg of the original IAM.SPH material was suspended in 7.0 mL of methanol. The resulting slurry was packed into the column at 60 MPa using a Haskel air-driven pump (Burbank, CA, USA), yielding the final working column. The mobile phase composition followed the protocol described by Russo et al. [31] and consisted of Dulbecco’s Phosphate Buffered Saline (DPBS), methanol, and acetonitrile in a 60:25:15 volume ratio. All solvents were of HPLC grade. DPBS contained 2.7 mmol·L^-1^ potassium chloride, 1.5 mmol·L^-1^ potassium dihydrogen phosphate, 137.0 mmol·L^-1^ sodium chloride and 8.1 mmol·L^-1^ disodium hydrogen phosphate (Merck). One PBS tablet was dissolved in 200 mL of Milli-Q water (18.2 MΩ·cm^-1^) under magnetic stirring. The pH of the buffer was determined using a calibrated pH meter (standards pH 4.0 and 7.0) and adjusted to 7.4 ± 0.05 with sodium hydroxide if required. The final mobile phase was vacuum filtered through a 0.2 μm nylon membrane prior to use in the HPLC system. Chromatographic analyses were performed on an Agilent 1260 HPLC system (Santa Clara, CA, USA) equipped with a binary pump, micro vacuum degasser, column thermostat, and autosampler. Detection was carried out using an Agilent 1260 Diode Array Detector set at 210, 220, 230, 260, 280 and 300 nm. The separation was performed at 25 °C with a flow rate of 300 μL·min^-1^ and an injection volume of 10 μL. Each sample was analysed in triplicate, and a blank injection was run after every nine samples. The standard run time was 30 minutes; however, if compounds were not detected, a second run of up to 2 hours was conducted. Reported results represent the average of triplicate measurements. The retention times of the investigated bisphenols and reference substances are listed in Tables S2 and S3 in the Supplementary material.

### Theoretical descriptors

**Figure 1.**
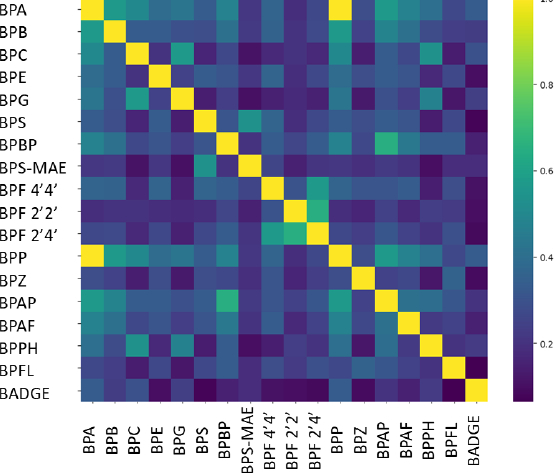
Heatmap of Tanimoto similarity indices for the investigated library of bisphenol derivatives

Utilizing Chemicalize (accessed on 1 June 2024) and the ACDlabs Percepta (version 2024.1.3) module, a series of lipophilicity indices for 18 bisphenol analogues was calculated and presented in Table 1. In addition, to provide further information about the studied molecules, physicochemical parameters such as ionization, water solubility, topological surface area and geometrical properties have been calculated using both programs and listed in Table S4. Furthermore, the extended connectivity fingerprint (ECFP4) with a radius of 2 and a bit vector length of 2048 bits was calculated using the RDKit package (version 2025.3.6) in Python. Following this, the pairwise Tanimoto similarity (ranging from 0 to 1) for the 18 bisphenol analogues under investigation was computed and visualized as a heatmap (Figure 1) to highlight similarities and differences.

**Table 1.** Theoretical lipophilicity indices together with chromatographically measured properties of the investigated bisphenol’s

Name	Calculated lipophilicity indices						Chromatographically determined parameters		
	ACD log *P* classic	Consensus log *P* ACD	ACD log *P* galas	ACD log *D*	log *P* chemicalize	log *D c*hemicalize	CHI_C18_	CHI_IAM_	log *k*^IAM.SPH^
BPA	3.43	3.50	3.52	3.50	4.05	4.04	72.12	41.05	0.89
BPB	3.96	3.93	3.91	3.93	4.49	4.49	79.12	44.60	1.13
BPC	4.35	4.25	4.18	4.25	5.07	5.07	84.86	45.77	1.22
BPE	3.08	3.04	3.02	3.04	3.75	3.74	67.33	38.75	0.75
BPG	6.11	5.62	5.21	5.62	6.54	6.53	107.67	53.28	N.D
BPS	1.83	1.81	1.80	1.74	2.32	1.98	45.20	30.23	0.09
BPBP	5.84	5.40	5.18	5.40	6.31	6.31	94.81	51.91	N.D
BPS-MAE	3.14	2.86	2.71	2.57	3.20	3.03	76.47	38.02	0.61
BPF 4’4’	2.73	2.96	3.02	2.96	3.46	3.45	61.54	36.52	0.53
BPF 2’2’	2.73	2.96	3.02	2.96	3.46	3.33	75.46	41.18	0.68
BPF 2’4’	2.73	2.96	3.02	2.96	3.46	3.45	66.48	38.22	0.59
BPP	6.12	5.92	5.62	5.92	6.72	6.72	101.98	55.01	2.06
BPZ	4.53	4.25	4.07	4.25	4.92	4.91	86.51	51.17	1.36
BPAP	4.57	4.38	4.20	4.38	5.18	5.17	83.96	47.21	1.38
BPAF	2.82	3.39	4.06	3.37	4.77	4.75	84.56	47.84	1.40
BPPH	6.35	5.87	5.31	5.87	7.34	7.34	108.87	56.05	N.D
BPFL	4.79	5.09	5.40	5.09	5.99	5.98	89.69	49.82	1.64
BADGE	3.95	3.53	3.21	3.53	4.02	4.02	102.16	41.13	1.36

N.D - not determined as it was not eluted

### Toxicity data and data analysis

Three distinct toxicity endpoints were selected to represent cellular, tissue-level, and organism-level effects and were chosen based on the availability of consistent and reliable experimental data. These included: (i) *in vitro* cytotoxicity determined in four mammalian cell lines (3T3-L1, MCF-7, C6, and HeLa), originally reported as IC_50_ / μM values from published sources [6]; (ii) *ex vivo* vasodilation measured as the percentage of maximal arterial relaxation [32]; and (iii) acute aquatic toxicity towards *Daphnia* spp., expressed as EC_50_ for immobilization from ECHA - REACH Registered Substances Database.

For correlation analyses, cytotoxicity data were transformed to potency-based endpoints according to pIC_50_ = -log_10_(IC_50_ / M). Aquatic toxicity values, originally reported in mg L^-1^, were converted to molar units using molecular weights and subsequently transformed to pEC_50_ using the same -log_10_ convention. For right-censored cytotoxicity values reported as IC_50_ >100 μM, three alternative treatments were considered during data preprocessing: assignment of a conservative value of 100 μM, exclusion of censored values, and assignment of an upper-bound value of 200 μM. The robustness of correlation results with respect to censoring treatment was evaluated using rank-based correlation analysis. Results reported in the main manuscript are based on the transformed dataset assuming IC_50_ = 200 μM for censored values, while alternative censoring scenarios are documented in the Supplementary material in Table S6. The completely transformed dataset used for correlation analyses is summarized in Table 2, while details of raw data, endpoint transformation, and sensitivity analyses are provided in the Supplementary material in Tables S5 and S6.

**Table 2.** Summary of cellular, tissue-level, and organism-level toxicity endpoints for the investigated bisphenol analogues

Name	pIC_50_ 3T3-L1	pIC_50_ MCF-7	pIC_50_ C6	pIC_50_ HeLa	Vasodilation %	pEC_50_ *Daphnia* spp
BPA	3.70	4.30	3.80	3.68	55.69	4.36
BPB	4.27	4.19	3.93	3.88	66.18	4.71
BPC	N.A.	N.A..	N.A.	N.A.	69.96	4.74
BPE	3.95	3.70	3.84	3.70	48.24	4.07
BPG	N.A.	N.A.	N.A.	N.A.	25.66	N.A.
BPS	3.70	3.70	3.77	3.52	24.65	3.66
BPBP	N.A.	N.A.	N.A.	N.A.	17.28	N.A.
BPS-MAE	N.A.	N.A.	N.A.	N.A.	N.A.	3.84
BPF 4’4’	3.96	3.70	3.62	3.56	41.49	N.A.
BPF 2’2’	N.A.	N.A.	N.A.	N.A.	N.A.	N.A.
BPF 2’4’	N.A.	N.A.	N.A.	N.A.	N.A.	N.A.
BPP	N.A.	N.A.	N.A.	N.A.	18.61	4.93
BPZ	N.A.	N.A.	N.A.	N.A.	27.53	N.A.
BPAP	N.A.	N.A.	N.A.	N.A.	80.73	N.A.
BPAF	4.94	4.44	4.35	4.23	83.14	4.09
BPPH	N.A.	N.A.	N.A.	N.A.	26.01	N.A.
BPFL	N.A.	N.A.	N.A.	N.A.	N.A.	N.A.
BADGE	4.15	4.69	4.04	3.98	N.A.	N.A.

N.A. - not available IC_50_ values exceeding 100 μM were truncated to 200 μM

To evaluate the relationship between lipophilicity and toxicity-related endpoints across different biological levels, correlation analyses were performed between each toxicity endpoint and the full set of chromatographic and theoretical descriptors. All analyses were conducted using potency-transformed toxicity endpoints (pIC_50_ for cytotoxicity and pEC_50_ for aquatic toxicity), as described above, to ensure consistent correlation directionality and biological interpretability. Both Pearson correlation coefficients (*r*) and Spearman rank correlation coefficients (*ρ*) were calculated. Pearson correlations were used to assess linear associations, following visual inspection of scatterplots, while Spearman correlations were employed as a rank-based, non-parametric measure robust to deviations from linearity and the presence of censored values.

For the main analysis presented in the manuscript, right-censored cytotoxicity values originally reported as IC_50_ > 100 μM were conservatively assigned a value of 200 μM prior to transformation. The robustness of correlation patterns with respect to alternative censoring treatments (100 μM, exclusion) was evaluated separately and is documented in Table S6. Missing toxicity or chromatographic values were handled using pairwise deletion. For the vasodilation endpoint, the relationship with CHI_IAM_ was further examined using a structured modelling approach to formally account for the visually apparent two-regime pattern. Pearson and Spearman correlations were calculated separately for the full dataset (*n* = 13) and the low-to-moderate CH_IIAM_ subset (*n* = 8). Three regression models were fitted to the full dataset: a simple linear model, a quadratic model, and a segmented (piecewise) regression model with an empirically estimated breakpoint, using the segmented package in *R*. Model fit was compared using adjusted *R*^2^, AIC, BIC, and residual standard error, and the quadratic model was additionally tested against the linear model by an F-test. Cook's distance was calculated for each observation (threshold: 4/*n*) to provide an objective assessment of individual compound influence. Full model comparison statistics and residual diagnostics are provided in the Supplementary material (Table S7). The resulting correlation matrices for all toxicity endpoints, together with correlation coefficients derived from the best-fitting model for the vasodilation endpoint, are presented in Table 3.

**Table 3.** Correlation of toxicity endpoints with chromatographic and computed lipophilicity descriptors

toxicity endpoint	ACD log *P* classic	Consensus log *P* ACD	ACD log *P* galas	ACD log *D*	log *P* chemicalize	log *D* chemicalize	CHI_C18_	CHI_IAM_	log *k*^IAM.SPH^
pIC_50_ 3T3-L1 *n*=7	*r*=0.20 *ρ*=0.36	*r*=0.45 *ρ*=0.50	*r*=0.69 *ρ*=0.71	*r*=0.45 *ρ*=0.50	*r*=0.73 *ρ*=0.68	*r*=0.69 *ρ*=0.70	*r*=0.56 *ρ*=0.70	***r*=0.79** ***ρ*=0.81**	*r*=0.73 *ρ*=0.77
pIC_50_ MCF-7 *n*=7	*r*=0.66 *ρ*=0.55	*r*=0.66 *ρ*=0.70	*r*=0.61 *ρ*=0.71	*r*=0.65 *ρ*=0.70	*r*=0.67 *ρ*=0.70	*r*=0.65 *ρ*=0.70	***r*=0.89** **ρ=0.92**	*r*=0.70 *ρ*=0.77	*r*=0.86 *ρ*=0.89
pIC_50_ C6 *n*=7	*r*=0.23 *ρ*=0.54	*r*=0.39 *ρ*=0.64	*r*=0.58 *ρ*=0.74	*r*=0.38 *ρ*=0.64	*r*=0.66 *ρ*=0.78	*r*=0.61 *ρ*=0.78	*r*=0.65 *ρ*=0.89	*r*=0.75 *ρ*=0.89	***r*=0.77** ***ρ*=0.92**
pIC_50_ HeLa *n*=7	*r*=0.44 *ρ*=0.57	*r*=0.60 *ρ*=0.68	*r*=0.74 *ρ*=0.79	*r*=0.59 *ρ*=0.68	*r*=0.81 *ρ*=0.82	*r*=0.78 *ρ*=0.82	*r*=0.79 *ρ*=0.93	*r*=0.87 *ρ*=0.93	***r*=0.90** ***ρ*=0.96**
Vasodilation* n=8	*r*=0.73 *ρ*=0.64	*r*=0.85 *ρ*=0.76	*r*=0.95 *ρ*=0.93	*r*=0.85 *ρ*=0.76	*r*=0.96 *ρ*=0.93	*r*=0.95 *ρ*=0.93	*r*=0.97 *ρ*=0.93	***r*=0.99** ***ρ*=1.00**	***r*=0.99 *ρ*=1.00**
pEC_50_ *Daphnia* spp *n*=8	*r*=0.73 *ρ*=0.64	*r*=0.83 *ρ*=0.83	*r*=0.92 *ρ*=0.95	*r*=0.82 *ρ*=0.83	*r*=0.90 *ρ*=0.95	*r*=0.90 *ρ*=0.95	*r*=0.93 *ρ*=0.95	***r*=0.97** ***ρ*=0.98**	*r*=0.94 *ρ*=0.98

*BPZ, BPG, BPP, BPPH, BPZ and BPBP were identified as non-linear with respect to the linear trends and were therefore excluded from the correlation analyses.

## Results and discussion

### Physicochemical and structural overview

To support the interpretation of the chromatographic and toxicity data, the bisphenol analogues were characterized with respect to their physicochemical and structural features. Generally, the investigated compounds exhibit a broad spectrum of physicochemical properties. At physiological pH, most of these molecules remain predominantly neutral. However, sulfone-containing analogues, such as BPS and its ether derivative, demonstrate measurable deprotonation, which is reflected in their increased aqueous solubility and reduced lipophilicity. Within this series, the calculated log *D*_7.4_ values range from approximately 1.7 to 6.0, aligning with experimentally determined properties, as the CHI_C18_ values indicate a lipophilicity interval of 45 to 110. The library displays significant variation in size, shape, and polar surface exposure. Bulky, polyaromatic structures, such as BPP, BPPH Rand BPFL, contrast with more polar or partially ionized members, including BPS, BPS-MAE and BADGE. Structural diversity is clearly illustrated in the heatmap in Figure 1, which shows Tanimoto similarity computed from ECFP fingerprints. Pairwise similarities are predominantly low to moderate, forming discrete clusters of closely related analogues rather than a single dense cluster. BPS and BADGE are chemically distinct from most bisphenols, consistent with their atypical linkers and functional groups. This dispersion in fingerprint space indicates that the dataset encompasses multiple chemotypes rather than minor substitutions on a common scaffold.

### Biomimetic chromatography

This study examined two phospholipid-modified stationary phases: one coated with phosphatidylcholine analogues, the predominant phospholipid in cell membranes, and the other with sphingomyelin. To facilitate comparison with traditional lipophilicity, a conventional C18 phase was also employed for the chromategraphic determination of bisphenols' lipophilicity. It should be emphasized that the bisphenols investigated in this study are, in the vast majority of cases, neutral molecules at physiological pH. Only two compounds in the dataset (BPS and BPS-MAE) show partial ionisation, and both are among the least lipophilic derivatives. Under these conditions, retention on membrane-mimetic phases primarily reflects lipophilicity-related interactions, while ionisation-related effects cannot be evaluated independently from overall hydrophilicity within the present compound set.

The initial significant observation pertains to the retention of analytes on the sphingomyelin column. Three highly lipophilic bisphenols (BPPH, BPBP and BPG) did not elute from the sphingomyelin column within the analytical timeframe. This is consistent with their extensive polyaromatic structures, substantial van der Waals volumes, and higher log *D* values, which facilitate strong interactions with sphingomyelin. This observation aligns with the pronounced retention of BPP (*t*_R_ ≈ 116.0 min; log *k*^IAM.SPH^ ≈ 2.06). Such behaviour of lipophilic bisphenols highlights the limitations of this chromatographic system. The exceedingly long retention time makes it impractical for high-throughput screening. The disparity between the sphingomyelin- and phosphatidylcholine-coated columns arises from the employment of different separation methodologies. To prevent damage to the sphingomyelin phase caused by a high concentration of organic modifier, isocratic separation was employed, with a maximum of 40 % organic modifier. In contrast, gradient elution was used for IAM, with the maximum ACN level reaching 85 %. Consequently, IAM operated under the Valkó gradient protocol facilitates rapid (~6.5 min *per* analysis) [33-34], reproducible CHI_IAM_ determination [35], extends the usable retention range, and allows elution of strongly hydrophobic bisphenols. Notably, for the subset of seven bisphenols previously measured on IAM under isocratic conditions, CHI_IAM_ values obtained with the Valkó gradient protocol exhibit a strong, practically linear correlation with the corresponding isocratic log *k*_IAM/w_ (*r* = 0.98, *p*-value = 1.2×10^-4^, *n* = 7) [6]. This suggests that IAM chromatography yields highly reproducible and robust results, including consistency in inter-laboratory comparisons.

Further noteworthy observations emerge from the BPF positional isomers. Across sphingomyelin, IAM, and C_18_, these isomers consistently exhibit the same retention order (2,2′, 2,4′, 4,4′), which aligns with the notion that ortho substitution facilitates intramolecular hydrogen bonding and steric shielding of phenolic polarity, thereby enhancing apparent membrane affinity. This effect is most pronounced on the C_18_ bonded column. In contrast, computational descriptors reveal nearly identical log *P* and log *D*_7.4_ values for these isomers across tested software, with only log *D* Chemicalize distinguishing BPF 2,2′ from the other two isomers. Their consistent separation across all three phases is attributed to orientation-dependent exposure of polar functionality and lipid-specific interactions, which are not captured by theoretical lipophilicity indices.

This highlights the importance of biomimetic chromatographic indices as an orthogonal input to IATA. By capturing subtle structure-interaction relationships that extend beyond traditional descriptors such as log *P*, IAM-derived parameters can enhance NAM-based hazard assessment by improving the interpretation of compound-membrane interactions and predictive modelling of toxicological endpoints. Notably, the distinct chromatographic behaviour of BPF isomers parallels known differences in their biological potencies, emphasizing the necessity for individualized evaluation of structural analogs in regulatory safety assessment [36].

### Correlation with toxicity data

To comprehensively evaluate the efficacy of biomimetic chromatography in predicting the toxic potency of bisphenol analogs, we selected three endpoints a *priori* based on their mechanistic association with membrane partitioning and their representation of distinct levels of biological organization and exposure: (*i*) *in vitro* cytotoxicity in mammalian cells [6], which captures cell entry and baseline narcosis-like effects; (*ii*) cardiotoxicity quantified as *ex vivo* vasodilation, a tissue-level functional readout sensitive to lipid-bilayer composition and modulation of membrane-embedded ion channels [33]; and (*iii*) aquatic toxicity towards Daphnids measured as EC_50_ for immobilization (OECD TG 202), the latter as organism-level endpoint integrating epithelial uptake and whole-animal narcosis. All selected endpoints thus capture toxicity mechanisms directly or indirectly influenced by compound-membrane interactions, making them particularly suitable for evaluating the translational value of biochromatographic indices. Given that some analogs exhibit partial ionization at physiological pH, both log *P* and log *D* were considered. All investigated toxicity data are compiled in Table 2, while the obtained correlations are listed in Table 3.

To ensure biological breadth and mechanistic contrast, we selected four mammalian cell lines that differ in tissue origin and receptor context: 3T3-L1 (adipocyte-like), MCF-7 (ER-positive breast epithelial), C6 (glial), and HeLa (epithelial, ER-negative), thereby enabling discrimination between receptor-modulated responses and receptor-independent, membrane-driven effects. Building on the findings of Russo and colleagues [6], which demonstrated a linear correlation between phospholipid affinity and in vitro cytotoxicity, this study aims to evaluate the applicability of fast-gradient protocols for predicting cytotoxicity. Furthermore, we seek to compare two distinct phospholipid-binding stationary phases and lipophilicity indices, both theoretical and chromatographically determined.

Consistent with previous observations, we found that membrane affinity correlates more closely with *in vitro* cytotoxicity. Furthermore, the concordance between Pearson’s linear correlation coefficients and Spearman’s rank-based correlations confirms that the reported associations are not driven by individual outliers and remain robust despite the relatively small dataset, with consistent trends observed across all applied data-handling strategies (Table S6). Notably, for each investigated cytotoxicity endpoint, chromategraphically determined parameters significantly exceeded calculated values, with the correlation coefficient approximately 0.1 higher than that of the best theoretical lipophilicity indices. Across the four cell lines, the concordant associations between toxicity and membrane-affinity metrics point to a predominantly non-specific, baseline toxicity mechanism, consistent with lipophilic partitioning and membrane perturbation rather than receptor-specific effects. In two out of the four tested cell lines, the highest value was observed for the log *k*^IAM.SPH^ parameter. However, it is crucial to consider the limitations of this method, particularly the extended analysis time and the inability to analyse highly lipophilic compounds.

**Figure 2.**
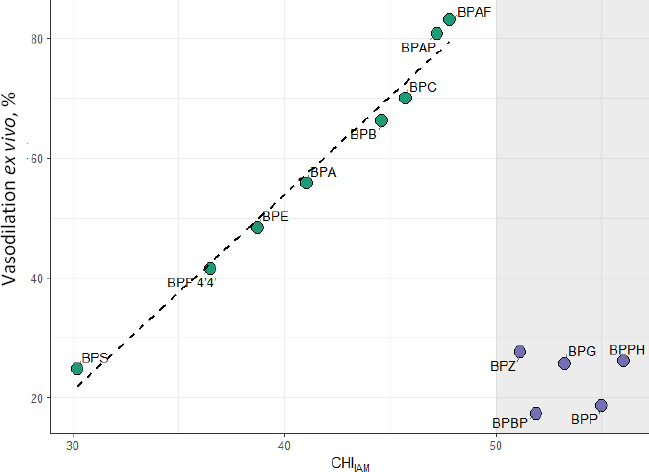
Relationship between CHI_IAM_ and bisphenol-induced vasodilation effect. The dashed line represents the linear trend for low-to-moderate CHI_IAM_ values, while compounds on the shaded background define a high-affinity regime deviating from this relationship

Given the well-established role of membrane interactions in regulating vascular tone, we further examined the relationship between chromatographically derived indices and *ex vivo* vasodilation data. This endpoint, which reflects tissue-level functional responses such as arterial relaxation, provides a mechanistic link between membrane affinity and potential cardiovascular toxicity. A noteworthy observation emerged from this investigation. Generally, there is a strong, nearly linear correlation between both lipophilicity and affinity for the artificial membrane, and the percentage of maximal vasodilation measured *ex vivo*. This suggests that these factors are key determinants of the vascular effects induced by bisphenol analogs. Compounds with higher phospholipid affinity demonstrated greater vasodilatory potency, indicating a potential role of membrane interactions in modulating vascular smooth muscle function. However, for the four compounds with the highest membrane affinity (BPZ, BPG, BPP, BPPH and BPBP), this trend significantly declined, with maximal vasodilation decreasing despite increasing lipophilicity/membrane affinity (Figure 2). Rather than representing random outliers, these compounds define a distinct high-affinity regime, characterised by a near-linear increase in vasodilation for low-to-moderate CHI_IAM_ values and a pronounced non-linear response at higher membrane affinity. To formally assess this two-regime pattern, four analytical scenarios were evaluated: a simple linear model fitted to all compounds, a quadratic model, a segmented model, and a restricted linear model applied to the low-to-moderate CHI_IAM_ subset (*n* = 8). The segmented model yielded an empirically estimated breakpoint at CHI_IAM_ = 47.21, achieving the best overall fit among all tested models (*R*^2^_adj_ = 0.741); full model comparison statistics are presented in Table S7, and the fitted curves are illustrated in Figure S1. Notably, this statistically derived threshold closely corresponds to the CHI_IAM_ = 50 boundary applied in the correlation analyses, providing independent quantitative support for the two-regime classification adopted in this study. Influence diagnostics further confirmed that none of the high-affinity compounds exceeded the Cook's distance threshold (4/*n* = 0.308), indicating that their separation reflects a structured mechanistic pattern rather than statistical leverage (Table S8). This observation may suggest that excessive membrane binding limits the ability of these compounds to reach the site of action within the vascular smooth muscle, possibly due to sequestration within the lipid bilayer. Importantly, because cytotoxicity data for these four outliers were lacking across all four cell lines, we could not verify whether the same attenuation occurs at the cellular level or contributes to overall cytotoxicity. As a whole, the data point to an optimal window of membrane affinity beyond which vascular effects are attenuated, underscoring the limits of lipophilicity alone for predicting tissue-level toxicity and highlighting the added mechanistic value of biomimetic chromatography.

The final aspect investigated was the evaluation of the correlation between the obtained data and the toxicity against aquatic organisms. Previous studies indicate that biomimetic chromatography, particularly with IAM columns, is a reliable predictor of aquatic toxicity across various trophic levels [23,37]. The literature demonstrates its potential applicability to both a diverse group of pharmaceuticals [38] and structurally similar UV filters [17].

In the context of bisphenols, as in prior toxicological assessments, the chromatographic data show the strongest correlations, with the highest observed for CHI_C18_.

Collectively, the presented results demonstrate that biomimetic chromatography offers a more precise and mechanistically grounded assessment of bisphenol toxicity than purely computational approaches. Across cellular, tissue, and organismal endpoints, chromatographically derived lipophilicity indices consistently outperformed theoretical descriptors, underscoring the central role of membrane affinity in shaping toxic potency. Although certain computational algorithms, particularly Chemicalize log *P*, yielded comparatively strong correlations, others showed limited predictive value, highlighting the methodological variability inherent to *in silico* estimations. By contrast, chromatographic indices exhibited stable and robust performance across all endpoints, each demonstrating high predictive potential. Building on this clear performance advantage, the chromatographic approach also provides practical benefits over the classical shake-flask method, that are a) it is inherently more suitable for highly lipophilic analytes, b) it enables reliable quantification of labile compounds through rapid on-column partitioning and separation of degradation products when the HPLC is connected with mass spectrometer, and c) it offers a faster, more cost-efficient, and solvent-saving workflow. Taken together, these findings position biomimetic chromatography as a powerful experimental platform that not only improves predictive accuracy but also enhances mechanistic interpretation, thereby strengthening its value within contemporary IATA-oriented toxicity assessment frameworks.

## Conclusions

This study demonstrates that biomimetic chromatography provides a robust, reproducible, and mechanistically informative measure of bisphenol lipophilicity, consistently outperforming computational predictions across cellular, tissue, and organismal toxicity endpoints. Nevertheless, for aquatic toxicity towards Daphnia spp. and the vasodilation endpoint, it should be acknowledged that selected in silico descriptors also exhibited comparatively strong performance, particularly Chemicalize log *P* and ACD log *P* Galas, which showed correlations approaching those obtained with chromatographically derived indices.

The chromatographic indices captured subtle structural effects, such as positional isomerism and lipid-specific interactions, that were not reflected in conventional in silico descriptors, underscoring a key mechanistic advantage of biomimetic chromatography. These results underscore the added value of experimentally derived membrane-affinity parameters in elucidating the physicochemical drivers of toxicity.

Importantly, the findings position biomimetic chromatography as a practical and scientifically grounded component of NAMs and an informative entry point within IATAs. By generating human-relevant, non-animal data on compound-membrane interactions, this technique enhances the predictive accuracy of hazard assessment, supports mechanistic interpretation of toxic effects, and contributes to the refinement and integration of alternative testing strategies for bisphenol analogs and structurally related environmental contaminants. Despite the limited overlap between individual toxicity endpoints, the observed relationships with lipophilicity-related descriptors consistently indicate that a substantial component of bisphenol toxicity is governed by lipophilicity-driven, membrane-related mechanisms. Moreover, biomimetic chromatography can be applied not only to human health-related endpoints but also to ecotoxicological assessments, providing a versatile framework for evaluating chemical safety across biological systems.

## Supplementary material

Additional data are available at https://pub.iapchem.org/ojs/index.php/admet/article/view/3169, or from the corresponding author on request.



## References

[1] Mishra A. Goel D. Shankar S. Bisphenol A contamination in aquatic environments: a review of sources, environmental concerns, and microbial remediation. Environmental Monitoring and Assessment 195 (2023) 1352. https://doi.org/10.1007/s10661-023-11977-1 10.1007/s10661-023-11977-1 37861868

[2] Pelch K.E. Li Y. Perera L. Thayer K.A. Korach K.S. Characterization of Estrogenic and Androgenic Activities for Bisphenol A-like Chemicals (BPs): *In Vitro* Estrogen and Androgen Receptors Transcriptional Activation, Gene Regulation, and Binding Profiles. Toxicological Sciences 172 (2019) 23-37. https://doi.org/10.1093/toxsci/kfz173 10.1093/toxsci/kfz173 31388671 PMC6813750

[3] Maniradhan M. Calivarathan L. Bisphenol A-Induced Endocrine Dysfunction and its Associated Metabolic Disorders. Endocrine, Metabolic & Immune Disorders - Drug Targets 23 (2023) 515-529. https://doi.org/10.2174/1871530322666220928144043 10.2174/1871530322666220928144043 36173044

[4] Khan N.G. Correia J. Adiga D. Rai P.S. Dsouza H.S. Chakrabarty S. Kabekkodu S.P. A comprehensive review on the carcinogenic potential of bisphenol A: clues and evidence. Environmental Science and Pollution Research 28 (2021) 19643-19663. https://doi.org/10.1007/s11356-021-13071-w 10.1007/s11356-021-13071-w 33666848 PMC8099816

[5] Adamovsky O. Groh K.J. Białk-Bielińska A. Escher B.I. Beaudouin R. Lagares L.M. Tollefsen K.E. Fenske M. Mulkiewicz E. Creusot N. Sosnowska A. Loureiro S. Beyer J. Repetto G. Štern A. Lopes I. Monteiro M. Zikova-Kloas A. Eleršek T. Vračko M. Zdybel S. Puzyn T. Koczur W. Morthorst J.E. Holbech H. Carlsson G. Örn S. Herrero Ó. Siddique A. Liess M. Braun G. Srebny V. Žegura B. Hinfray N. Brion F. Knapen D. Vandeputte E. Stinckens E. Vergauwen L. Behrendt L. Silva M.J. Blaha L. Kyriakopoulou K. Exploring BPA alternatives - Environmental levels and toxicity review. Environment International 189 (2024) 108728. https://doi.org/10.1016/j.envint.2024.108728 10.1016/j.envint.2024.108728 38850672

[6] Russo G. Capuozzo A. Barbato F. Irace C. Santamaria R. Grumetto L. Cytotoxicity of seven bisphenol analogues compared to bisphenol A and relationships with membrane affinity data. Chemosphere 201 (2018) 432-440. https://doi.org/10.1016/j.chemosphere.2018.03.014 10.1016/j.chemosphere.2018.03.014 29529570

[7] Sheng Q.-S. Liu B. Wang X. Hua L. Zhao S.-C. Sun X.-Z. Li M.-Y. Zhang X.-Y. Wang J.-X. Hu P.-L. Revolutionizing toxicological risk assessment: integrative advances in new approach methodologies (NAMs) and precision toxicology. Archives of Toxicology 99 (2025) 4697-4707. https://doi.org/10.1007/s00204-025-04169-y 10.1007/s00204-025-04169-y 40892062

[8] Jaeschke H. Ramachandran A. Are New Approach Methodologies (NAMs) the Holy Grail of toxicology? Toxicological Sciences 208 (2025) 1-8. https://doi.org/10.1093/toxsci/kfaf113 10.1093/toxsci/kfaf113 40795217 PMC12790340

[9] OECD. Overview of Concepts and Available Guidance related to Integrated Approaches to Testing and Assessment (IATA). OECD Series on Testing and Assessment 329 (2020). https://doi.org/10.1787/cd920ca4-en 10.1787/cd920ca4-en

[10] Meyer H. Zur Theorie der Alkoholnarkose. Archiv für experimentelle Pathologie und Pharmakologie 42 (1899) 109-118. https://doi.org/10.1007/bf01834479 10.1007/bf01834479

[11] Hansch C. Steward A.R. Iwasa J. Deutsch E.W. The Use of a Hydrophobic Bonding Constant for Structure-Activity Correlations. Molecular Pharmacology 1 (1965) 205-213. https://doi.org/10.1016/S0026-895X(25)14764-0 10.1016/S0026-895X(25)14764-0 5842822

[12] Glave W.R. Hansch C. Relationship between Lipophilic Character and Anesthetic Activity. Journal of Pharmaceutical Sciences 61 (1972) 589-591. https://doi.org/10.1002/jps.2600610420 10.1002/jps.2600610420 5014317

[13] Pidgeon C. Venkataram U.V. Immobilized artificial membrane chromatography: Supports composed of membrane lipids. Analytical Biochemistry 176 (1989) 36-47. https://doi.org/10.1016/0003-2697(89)90269-8 10.1016/0003-2697(89)90269-8 2712289

[14] Valko K.L. Biomimetic chromatography-A novel application of the chromatographic principles. Analytical Sciences Advances 3 (2022) 146-153. https://doi.org/10.1002/ansa.202200004 10.1002/ansa.202200004 PMC10989578 38715641

[15] Valkó K.L. Lipophilicity and biomimetic properties measured by HPLC to support drug discovery. Journal of Pharmaceutical and Biomedical Analysis 130 (2016) 35-54. https://doi.org/10.1016/j.jpba.2016.04.009 10.1016/j.jpba.2016.04.009 27084527

[16] Bunnally S. Young R.J. The role and impact of high throughput biomimetic measurements in drug discovery. ADMET & DMPK 6 (2018) 74-84. https://doi.org/10.5599/admet.530 10.5599/admet.530

[17] Stergiopoulos C. Tsopelas F. Ochsenkühn-Petropoulou M. Valko K. Predicting the acute aquatic toxicity of organic UV filters used in cosmetic formulations. ADMET & DMPK 12 (2024) 781-796. https://doi.org/10.5599/admet.2364 10.5599/admet.2364 39524218 PMC11542717

[18] Stergiopoulos C. Tsopelas F. Valko K. Prediction of hERG inhibition of drug discovery compounds using biomimetic HPLC measurements. ADMET & DMPK 9 (2021) 191-207. https://doi.org/10.5599/admet.995 10.5599/admet.995 35300361 PMC8920097

[19] Russo G. Grumetto L. Szucs R. Barbato F. Lynen F. Screening therapeutics according to their uptake across the blood-brain barrier: A high throughput method based on immobilized artificial membrane liquid chromatography-diode-array-detection coupled to electrospray-time-of-flight mass spectrometry. European Journal of Pharmaceutics and Biopharmaceutics 127 (2018) 72-84. https://doi.org/10.1016/j.ejpb.2018.02.004 10.1016/j.ejpb.2018.02.004 29427629

[20] Kovačević S. Banjac M.K. Milošević N. Ćurčić J. Marjanović D. Todorović N. Krmar J. Podunavac-Kuzmanović S. Banjac N. Ušćumlić G. Comparative chemometric and quantitative structure-retention relationship analysis of anisotropic lipophilicity of 1-arylsuccinimide derivatives determined in high-performance thin-layer chromatography system with aprotic solvents. Journal of Chromatography A 1628 (2020) 461439. https://doi.org/10.1016/j.chroma.2020.461439 10.1016/j.chroma.2020.461439 32822979

[21] Neri I. Piccolo M. Russo G. Ferraro M.G. Marotta V. Santamaria R. Grumetto L. The combined use of biological investigations, bio chromatographic and in silico methods to solve the puzzle of badge and its derivative’s toxicity. Chemosphere 367 (2024) 143640. https://doi.org/10.1016/j.chemosphere.2024.143640 10.1016/j.chemosphere.2024.143640 39490425

[22] Tsopelas F. Stergiopoulos F. Tsantili-Kakoulidou A. Immobilized artificial membrane chromatography: from medicinal chemistry to environmental sciences. ADMET & DMPK 6 (2018) 225-241. https://doi.org/10.5599/admet.553 10.5599/admet.553

[23] Stergiopoulos C. Makarouni D. Tsantili-Kakoulidou A. Ochsenkühn-Petropoulou M. Tsopelas F. Immobilized artificial membrane chromatography as a tool for the prediction of ecotoxicity of pesticides. Chemosphere 224 (2019) 128-139. https://doi.org/10.1016/j.chemosphere.2019.02.075 10.1016/j.chemosphere.2019.02.075 30818191

[24] Sobańska A.W. Affinity of Compounds for Phosphatydylcholine-Based Immobilized Artificial Membrane—A Measure of Their Bioconcentration in Aquatic Organisms. Membranes 12 (2022) 1130. https://doi.org/10.3390/membranes12111130 10.3390/membranes12111130 36422122 PMC9692598

[25] Sobańska A.W. RP-18 TLC retention data and calculated physico-chemical parameters as predictors of soil-water partition and bioconcentration of organic sunscreens. Chemosphere 279 (2021) 130527. https://doi.org/10.1016/j.chemosphere.2021.130527 10.1016/j.chemosphere.2021.130527 33873066

[26] Sobańska A.W. Evaluation of drug-likeness and ADME properties of sunscreens and preservatives using reversed-phase thin layer chromatographic retention data and calculated descriptors. Journal of Pharmaceutical and Biomedical Analysis 201 (2021) 114126. https://doi.org/10.1016/j.jpba.2021.114126 10.1016/j.jpba.2021.114126 33989995

[27] Valko K.L. Zhang T. Biomimetic properties and estimated in vivo distribution of chloroquine and hydroxy-chloroquine enantiomers. ADMET & DMPK 9 (2021) 151-165. https://doi.org/10.5599/admet.929 10.5599/admet.929 35299770 PMC8920107

[28] Szulczyk D. Woziński M. Koliński M. Kmiecik S. Głogowska A. Augustynowicz-Kopeć E. Dobrowolski M.A. Roszkowski P. Struga M. Ciura K. Menthol- and thymol-based ciprofloxacin derivatives against *Mycobacterium tuberculosis*: in vitro activity, lipophilicity, and computational studies. Scientific Reports 13 (2023) 16328. https://doi.org/10.1038/s41598-023-43708-4 10.1038/s41598-023-43708-4 37770610 PMC10539350

[29] Ulenberg S. Ciura K. Georgiev P. Pastewska M. Ślifirski G. Król M. Herold F. Bączek T. Use of biomimetic chromatography and in vitro assay to develop predictive GA-MLR model for use in drug-property prediction among anti-depressant drug candidates. Microchemical Journal 175 (2022) 107183. https://doi.org/10.1016/j.microc.2022.107183 10.1016/j.microc.2022.107183

[30] Ciura K. Kovačević S. Pastewska M. Kapica H. Kornela M. Sawicki W. Prediction of the chromatographic hydrophobicity index with immobilized artificial membrane chromatography using simple molecular descriptors and artificial neural networks. Journal of Chromatography A 1660 (2021) 462666. https://doi.org/10.1016/j.chroma.2021.462666 10.1016/j.chroma.2021.462666 34781046

[31] Russo G. Ermondi G. Caron G. Verzele D. Lynen F. Into the first biomimetic sphingomyelin stationary phase: Suitability in drugs’ biopharmaceutic profiling and block relevance analysis of selectivity. European Journal of Pharmaceutical Sciences 156 (2021) 105585. https://doi.org/10.1016/j.ejps.2020.105585 10.1016/j.ejps.2020.105585 33045369

[32] Tvrdý V. Dias P. Nejmanová I. Carazo A. Jirkovský E. Pourová J. Fadraersada J. Moravcová M. Mašič L.P. Dolenc M.S. Mladěnka P. The effects of bisphenols on the cardiovascular system ex vivo and in vivo. Chemosphere 313 (2023) 137565. https://doi.org/10.1016/j.chemosphere.2022.137565 10.1016/j.chemosphere.2022.137565 36528156

[33] Valko K.L. Nunhuck S. Bevan C. Abraham M.H. Reynolds D.P. Fast gradient HPLC method to determine compounds binding to human serum albumin. Relationships with octanol/water and immobilized artificial membrane lipophilicity. Journal of Pharmaceutical Sciences 92 (2003) 2236-2248. https://doi.org/10.1002/jps.10494 10.1002/jps.10494 14603509

[34] Valko K.L. Rava S. Bunnally S. Anderson S. Revisiting the application of Immobilized Artificial Membrane (IAM) chromatography to estimate in vivo distribution properties of drug discovery compounds based on the model of marketed drugs. ADMET & DMPK 8 (2020) 78-97. https://doi.org/10.5599/admet.757 10.5599/admet.757 35299777 PMC8915595

[35] Ciura K. Modeling of small molecule affinity to phospholipids using IAM-HPLC and a QSRR approach enhanced by similarity-based machine learning algorithms. Journal of Chromatography A 1714 (2024) 464549. https://doi.org/10.1016/j.chroma.2023.464549 10.1016/j.chroma.2023.464549 38056392

[36] Matteo G. Leingartner K. Rowan-Carroll A. Meier M. Williams A. Beal M.A. Gagné M. Farmahin R. Wickramasuriya S. Reardon A.J.F. Barton-Maclaren T. Corton J.C. Yauk C.L. Atlas E. In vitro transcriptomic analyses reveal pathway perturbations, estrogenic activities, and potencies of data-poor BPA alternative chemicals. Toxicological Sciences 191 (2022) 266-275. https://doi.org/10.1093/toxsci/kfac127 10.1093/toxsci/kfac127 PMC9936204 36534918

[37] Stergiopoulos C. Tsakanika L.A. Ochsenkühn-Petropoulou M. Kakoulidou A.T. Tsopelas F. Application of micellar liquid chromatography to model ecotoxicity of pesticides. Comparison with immobilized artificial membrane chromatography and n-octanol-water partitioning. Journal of Chromatography A 1696 (2023) 463951. https://doi.org/10.1016/j.chroma.2023.463951 10.1016/j.chroma.2023.463951 37054635

[38] Stergiopoulos C. Tsopelas F. Ochsenkühn-Petropoulou M. Valko K. The use of biomimetic chromatography to predict acute aquatic toxicity of pharmaceutical compounds. Toxicology and Environmental Chemistry 104 (2022) 1-19. https://doi.org/10.1080/02772248.2021.2005065 10.1080/02772248.2021.2005065

