# Supplementary material for: Modelling an alternative lipophilicity scale of bisphenols using biomimetic chromatography: Relevance to membrane-driven baseline toxicity
Source: ADMET DMPK. 2026 Mar 19;14:3169. doi: 10.5599/admet.3169 (PMC13147520; doi:10.5599/admet.3169)
Supplement: Supplementary file 1 [file ADMET-14-3169-S1.pdf]

Open Access : ISSN : 1848-7718
http://www.pub.iapchem.org/ojs/index.php/admet

Supplementary material to

Modelling an alternative lipophilicity scale of bisphenols using biomimetic chromatography: Relevance to membrane-driven baseline toxicity

Krzesimir Ciura[1,2], Julia Nicman[1], Szymon Zdybel[2,3], Giacomo Russo[4], Lucia Grumetto[5], Katarzyna Ewa Greber[1], Anita Sosnowska[2,3], Joanna Dołżonek[6] and Karolina Jagiello[2,3]

[1]*Department of Physical Chemistry, Faculty of Pharmacy, Medical University of Gdańsk, Al. Gen. J. Hallera 107, 80-416, Gdańsk, Poland*
[2]*Laboratory of Environmental Chemoinformatics, Faculty of Chemistry, University of Gdansk, Wita Stwosza 63, 80-308, Gdansk, Poland*
[3]*QSAR Lab Ltd., Trzy Lipy 3 St., 80-172 Gdańsk, Poland*
[4]*School of Applied Sciences, Sighthill Campus, Edinburgh Napier University, 9 Sighthill Ct, EH11 4BN Edinburgh, United Kingdom*
[5]*Department of Pharmacy, School of Medicine and Surgery, University of Naples Federico II, Via D. Montesano, 49, 80131, Naples, Italy*
[6]*Department of Environmental Analysis, Faculty of Chemistry, University of Gdansk, Wita Stwosza 63, 80-308, Gdansk, Poland*

ADMET & DMPK **00(0)** (2026) 3169; https://doi.org/10.5599/admet.3169

Table S1. 2D structures of investigated BPA analogues

No.	Structural	Name	CAS
1	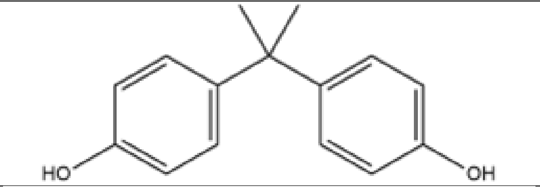	Bisphenol A (BPA)	80-05-7
2	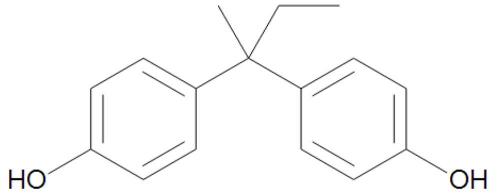	Bisphenol B (BPB)	77-40-7
3	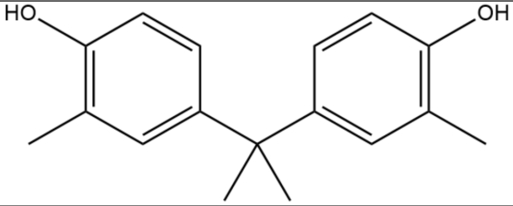	Bisphenol C (BPC)	79-97-0
4	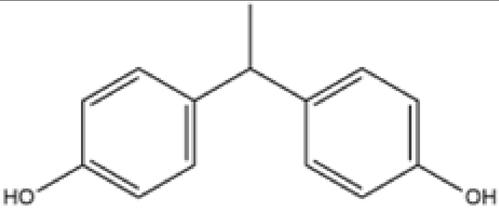	Bisphenol E (BPE)	2081-08-5

No.	Structural	Name	CAS
5	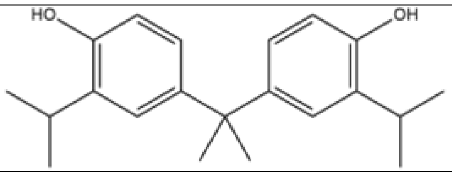	Bisphenol G (BPG)	127-54-8
6	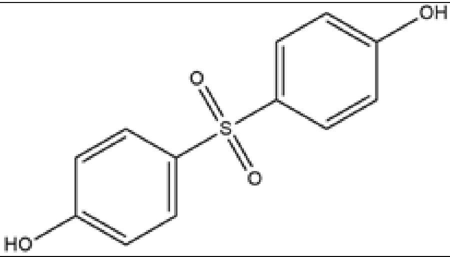	Bisphenol S (BPS)	80-09-1
7	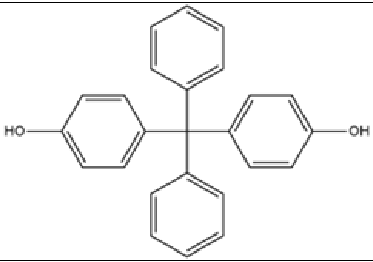	Bisphenol BP (BPBP)	1844-01-5
8	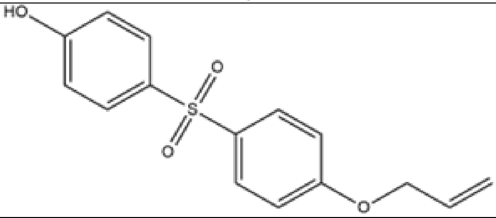	2,2-bis(4-hydroxyphenyl)-4-methylpentane (BPS MAE)	97042-18-7
9	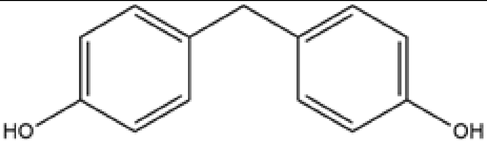	4,4'-bisphenol F (4,4'-BPF)	620-92-8
10	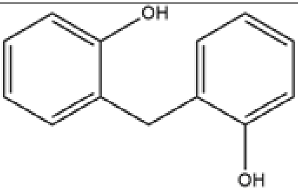	2,2'-bisphenol F (2,2'-BPF)	2467-02-9
11	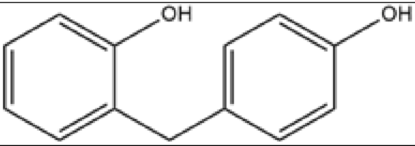	2,4'-bisphenol F (2,4'-BPF)	2467-03-0
12	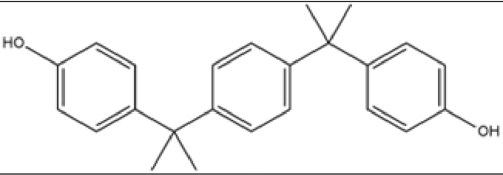	Bisphenol P (BPP)	2167-51-3
13	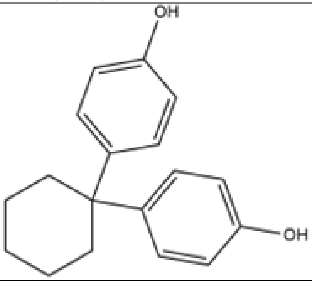	Bisphenol Z (BPZ)	843-55-0

CC BY

No.	Structural	Name	CAS
14	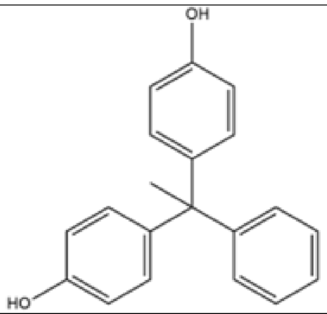	Bisphenol AP (BPAP)	1571-75-1
15	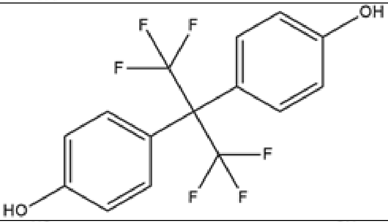	Bisphenol AF (BPAF)	1478-61-1
16	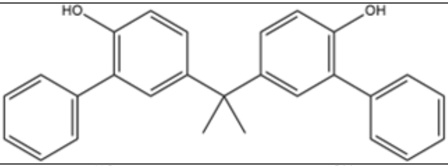	Bisphenol PH (BPPH)	24038-68-4
17	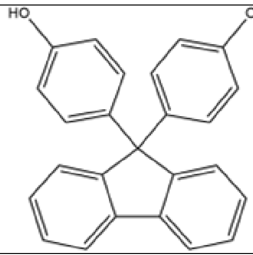	Bisphenol FL (BPFL)	3236-71-3
18	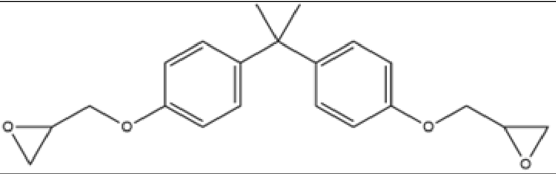	Bisphenol A diglycidyl ether (BADGE)	1675-54-3

Table S2. Retention times of bisphenol analogues determined by biomimetic liquid chromatography

	C18 at pH 7.4						IAM						IAM.SPH				
	Retention time, min					CHI_{C18}	Retention time, min					CHI_{IAM}	Retention time, min				SD
	t_1	t_2	t_3	t_{mean}	SD		t_1	t_2	t_3	t_{mean}	SD		t_1	t_2	t_3	t_{mean}	
BPA	4.644	4.643	4.651	4.646	0.004	72.124	5.144	5.129	5.133	5.135	0.008	41.045	8.174	8.206	8.214	8.198	0.021
BPB	4.880	4.877	4.888	4.882	0.006	79.119	5.393	5.370	5.375	5.379	0.012	44.600	13.496	13.496	13.530	13.507	0.020
BPC	5.074	5.075	5.076	5.075	0.001	84.858	5.465	5.455	5.459	5.460	0.005	45.771	16.535	16.497	16.512	16.515	0.019
BPE	4.484	4.486	4.483	4.484	0.002	67.326	4.979	4.976	4.978	4.978	0.002	38.748	6.200	6.199	6.202	6.200	0.002
BPG	5.842	5.842	5.847	5.844	0.003	107.673	5.974	5.975	5.976	5.975	0.001	53.279	–	–	–	–	–
BPS	3.735	3.742	3.740	3.739	0.004	45.203	4.389	4.394	4.397	4.393	0.004	30.235	2.106	2.095	2.101	2.101	0.006
BPBP	5.411	5.411	5.409	5.410	0.001	94.811	5.877	5.882	5.885	5.881	0.004	51.914	–	–	–	–	–
BPS-MAE	4.788	4.799	4.790	4.792	0.006	76.468	4.927	4.924	4.933	5.245	0.005	38.023	4.717	4.760	4.760	4.746	0.025
BPF 4′4′	4.282	4.298	4.288	4.289	0.008	61.538	4.830	4.819	4.826	4.825	0.006	36.524	4.139	4.174	4.177	4.163	0.021
BPF 2′2′	4.753	4.769	4.753	4.758	0.009	75.459	5.142	5.144	5.147	5.144	0.003	41.176	5.488	5.524	5.542	5.518	0.027
BPF 2′4′	4.449	4.462	4.457	4.456	0.007	66.485	4.942	4.941	4.942	4.942	0.001	38.224	4.613	4.602	4.624	4.613	0.011
BPP	5.646	5.657	5.653	5.652	0.006	101.984	6.090	6.102	6.090	6.094	0.007	55.013	110.007	110.006	110.006	110.006	0.001
BPZ	5.130	5.129	5.133	5.131	0.002	86.510	5.696	6.102	5.693	5.830	0.235	51.171	22.765	22.893	22.325	22.661	0.298
BPAP	5.044	5.043	5.047	5.045	0.002	83.958	5.554	5.565	5.556	5.558	0.006	47.208	23.228	23.529	23.806	23.521	0.289
BPAF	5.062	5.061	5.072	5.065	0.006	84.561	5.606	5.603	5.596	5.602	0.005	47.839	24.306	24.425	24.920	24.550	0.326
BPPH	5.881	5.886	5.885	5.884	0.003	108.871	6.170	6.163	6.163	6.165	0.004	56.052	–	–	–	–	–
BPFL	5.244	5.230	5.239	5.238	0.007	89.686	5.739	5.727	5.747	5.738	0.010	49.821	42.463	41.994	42.326	42.261	0.241
BADGE	5.662	5.655	5.657	5.658	0.004	102.163	5.144	5.137	5.142	5.141	0.004	41.128	22.351	22.598	22.533	22.494	0.128

t_1, t_2, t_3 - retention times from triplicate measurements; t_{mean} - mean retention time; SD - standard deviation; CHI_{C18} - chromatographic hydrophobicity index determined on C18 column; CHI_{IAM} - chromatographic hydrophobicity index determined on IAM column.

Table S3. Retention times of reference standards

Compound	C18 at pH 7.4					
	t_1 / min	t_2 / min	t_3 / min	t_{mean} / min	SD, min	CHI_{C18}
Theophylline	2.853	2.867	2.844	2.855	0.012	18.4
Benzimidazole	3.311	3.313	3.301	3.308	0.006	34.3
Colichicine	3.919	3.930	3.908	3.919	0.011	45
Acetophenone	4.215	4.214	4.205	4.211	0.006	65.1
Indole	4.570	4.560	4.560	4.563	0.006	71.5
Propiophenone	4.762	4.761	4.751	4.758	0.006	77.4
Butyrophenone	5.172	5.173	5.161	5.169	0.007	87.5
Valerophenone	5.541	5.543	5.530	5.538	0.007	96.2
				IAM		
Paracetamol	2.542	2.533	2.535	2.537	0.005	2.9
Acetanilidine	3.086	3.081	3.084	3.084	0.003	11.5
Acetophenone	3.476	3.471	3.472	3.473	0.003	17.2
Propiohenone	4.102	4.097	4.098	4.099	0.003	25.9
Butyrophenone	4.544	4.539	4.539	4.541	0.003	32.0
Valerophenone	4.905	4.902	4.901	4.903	0.002	37.3
Hexanophenone	5.196	5.193	5.193	5.194	0.002	41.8
Heptanophenone	5.458	5.455	5.454	5.456	0.002	45.7
Octanophenone	5.678	5.677	5.677	5.677	0.001	49.4

t_1, t_2, t_3- retention times from triplicate measurements; t_{mean} - mean retention time; SD - standard deviation; CHI_{C18} - chromatographic hydrophobicity index determined on C18 column; CHI_{IAM} - chromatographic hydrophobicity index determined on IAM column.

Table S4. Molecular descriptors implemented in the study

Name	SS_1	SS_2	SS_3	EP_1, Å^3	EP_2, cm^3·mol^{-1}	LH_1	SS_4, Å^3	SS_5, Å^2	SS_6, Å^2	SS_7, Å^2	SS_8, Å^2	SS_9, Å	SS_10, Å^2
BPA	33	17	0.20	26.59	79.28	5.64	221.41	359.72	413.10	43.37	67.22	4.37	6.28
BPB	36	18	0.25	28.43	83.88	5.24	238.57	390.67	426.24	44.78	70.47	4.78	6.18
BPC	39	19	0.29	30.12	89.36	4.85	255.30	423.84	492.76	45.74	75.37	4.52	6.69
BPE	30	16	0.14	24.74	64.31	6.06	203.89	325.42	404.66	38.56	61.29	4.25	6.33
BPG	51	23	0.43	37.50	107.66	3.39	323.62	544.41	571.94	51.24	90.58	4.60	7.31
BPS	27	17	0.00	25.60	63.55	9.14	205.42	328.08	428.23	44.00	64.79	4.34	6.53
BPBP	47	27	0.04	42.15	108.97	3.48	329.95	507.72	535.29	72.99	82.44	5.88	6.40
BPS MAE	34	20	0.07	30.95	77.20	8.21	249.58	396.57	506.91	46.34	76.23	4.65	7.55
4,4'-BPF	27	15	0.08	22.90	59.76	6.50	186.42	295.79	383.94	38.47	62.38	3.75	6.13
2,2'-BPF	27	15	0.08	22.90	59.76	6.50	186.57	295.49	359.94	38.85	62.12	4.12	5.49
2,4'-BPF	27	15	0.08	22.90	59.76	6.50	186.53	295.79	374.81	37.99	62.86	3.77	5.95
BPP	52	26	0.25	41.67	128.53	3.09	344.19	562.33	585.13	53.66	101.43	5.29	7.15
BPZ	40	20	0.33	31.39	91.28	4.49	261.32	420.67	426.71	53.91	73.55	4.96	6.23
BPAP	40	22	0.10	34.37	89.75	4.49	275.57	435.12	490.08	57.15	76.52	5.74	6.11
BPAF	33	23	0.20	25.44	81.14	5.08	251.62	391.64	416.34	48.88	70.09	4.72	6.20
BPPH	53	29	0.11	48.73	129.55	2.81	362.78	573.85	625.60	62.49	106.02	6.08	8.50
BPFL	45	27	0.04	42.82	108.05	3.49	316.58	483.86	517.99	69.15	82.46	6.09	6.34
BADGE	49	25	0.43	37.69	105.73	6.74	319.30	539.63	645.86	55.93	97.25	5.92	8.68

Name	JP_1	TD_1, kJ·mol^{-1}	TD_2, °C	LH_2	LH_4, log (mol·L^{-1})	LH_3	JP_3	JP_2
BPA	-0.01	67.72	192.42	3.63	6.70	3.73	1.00	0.00
BPB	-0.01	69.07	195.52	3.92	6.75	3.90	1.00	0.00
BPC	0.00	66.42	190.62	4.33	6.88	4.30	1.00	0.00
BPE	-0.01	64.77	181.38	2.98	6.46	2.94	1.00	0.00
BPG	0.00	70.31	184.64	5.62	6.95	5.22	1.00	0.00
BPS	-0.81	80.43	259.41	1.72	5.27	1.80	0.81	0.19
BPBP	-0.02	83.61	240.59	5.53	6.99	5.23	1.00	0.00
BPS MAE	-0.32	79.24	253.66	2.47	5.49	2.71	0.40	0.60
4,4'-BPF	-0.01	66.43	192.92	2.90	6.30	2.95	1.00	0.00
2,2'-BPF	-0.26	63.24	177.05	2.90	6.25	2.95	1.00	0.00
2,4'-BPF	-0.01	64.81	184.92	2.90	6.29	2.95	1.00	0.00
BPP	-0.01	81.63	230.08	5.92	6.99	5.62	1.00	0.00
BPZ	-0.01	72.50	207.34	4.14	6.58	3.79	0.99	0.01
BPAP	-0.02	76.52	221.68	4.39	6.95	4.22	1.00	0.00
BPAF	-0.06	61.13	161.93	3.45	6.81	4.44	0.96	0.04
BPPH	-0.01	88.34	252.82	5.95	7.00	5.55	1.00	0.00
BPFL	-0.02	83.09	241.00	5.07	7.00	5.38	0.99	0.01
BADGE	0.00	72.42	148.54	3.57	7.00	3.27	1.00	0.00

SS_1 - atom count; SS_2 - heavy atom count; SS_3 - fraction of sp^3 carbons (FSP3); EP_1 – polarizability; EP_2 - molar refractivity; LH_1 - hydrophilic-lipophilic balance (HLB); SS_4 - Van der Waals volume; SS_5 - Van der Waals surface area; SS_6 - solvent accessible surface area; SS_7 - minimum projection area; SS_8 - maximum projection area; SS_9 - minimum projection radius; SS_10 - maximum projection radius; JP_1 - isoelectric point charge; TD_1 - enthalpy of vaporization; TD_2 - flash point; LH_2 – logarithm of distribution coefficient (log D); LH_4 – logarithm of aqueous solubility, log S_w; LH_3 – logarithm of partition coefficient (log P); JP_3 - neutral form; JP_2 - negative form

(cc) BY

Table 5. Summary of cellular, tissue-level, and organism-level toxicity endpoints for the investigated bisphenol analogues

Name	IC_{50}, µM				Vasodilation, %	EC_{50} aquatic toxicity, mg·L^{-1}
	3T3-L1	MCF-7	C6	HeLa		
BPA	>100	50	160	209.1	55.69	9.94
BPB	53.8	64.4	118.5	130.5	66.18	4.72
BPC	N.A.	N.A.	N.A.	N.A.	69.96	4.7
BPE	112.5	>100	144.2	200.4	48.24	18
BPG	N.A.	N.A.	N.A.	N.A.	25.66	N.A.
BPS	>100	>100	168.4	299.3	24.65	55
BPBP	N.A.	N.A.	N.A.	N.A.	17.28	N.A.
BPS-MAE	N.A.	N.A.	N.A.	N.A.	N.A.	13.5
BPF 4’4’	110.6	>100	239.4	274.4	41.49	N.A.
BPF 2’2’	N.A.	N.A.	N.A.	N.A.	N.A.	N.A.
BPF 2’4’	N.A.	N.A.	N.A.	N.A.	N.A.	N.A.
BPP	N.A.	N.A.	N.A.	N.A.	18.61	1.6
BPZ	N.A.	N.A.	N.A.	N.A.	27.53	N.A.
BPAP	N.A.	N.A.	N.A.	N.A.	80.73	N.A.
BPAF	11.5	36.4	44.5	58.3	83.14	2.7
BPPH	N.A.	N.A.	N.A.	N.A.	26.01	N.A.
BPFL	N.A.	N.A.	N.A.	N.A.	N.A.	N.A.
BADGE	71	20.2	91.6	105.5	N.A.	N.A.

N.A. – not available

Table S6. Pearson correlation coefficients (r) and Spearman rank correlation coefficients (ρ) between lipophilicity descriptors and toxicity endpoints for the investigated bisphenol analogues. Right-censored IC_{50} values (>100 µM) were handled using three scenarios: (a) conservative assignment to 100 µM, (b) upper-bound assignment to 200 µM, (c) exclusion of censored observations

Endpoint	Descriptor	n	Scenario	r	ρ
3T3_L1	ACD_log *P*_classic	7	a	0.06	0.36
3T3_L1	Consensus_log *P*_ACD	7	a	0.34	0.58
3T3_L1	ACD_log *P*_galas	7	a	0.62	0.78
3T3_L1	ACD_log *D*	7	a	0.32	0.58
3T3_L1	log *P*_chemicalize	7	a	0.65	0.76
3T3_L1	log *D*_chemicalize	7	a	0.60	0.76
3T3_L1	CHI_{C18}	7	a	0.45	0.70
3T3_L1	CHI_{IAM}	7	a	0.74	0.81
3T3_L1	log *k*_IAM_SPH	7	a	0.65	0.77
MCF7	ACD_log *P*_classic	7	a	0.59	0.56
MCF7	Consensus_log *P*_ACD	7	a	0.55	0.70
MCF7	ACD_log *P*_galas	7	a	0.49	0.71
MCF7	ACD_log *D*	7	a	0.54	0.70
MCF7	log *P*_chemicalize	7	a	0.57	0.70
MCF7	log *D*_chemicalize	7	a	0.56	0.70
MCF7	CHI_{C18}	7	a	0.90	0.93
MCF7	CHI_{IAM}	7	a	0.59	0.78
MCF7	log *k*_IAM_SPH	7	a	0.82	0.89
C6	ACD_log *P*_classic	7	a	0.23	0.54
C6	Consensus_log *P*_ACD	7	a	0.39	0.64
C6	ACD_log *P*_galas	7	a	0.58	0.74
C6	ACD_log *D*	7	a	0.38	0.64
C6	log *P*_chemicalize	7	a	0.66	0.79
C6	log *D*_chemicalize	7	a	0.61	0.79
C6	CHI_{C18}	7	a	0.65	0.89
C6	CHI_{IAM}	7	a	0.75	0.89
C6	log *k*_IAM_SPH	7	a	0.77	0.93
HeLa	ACD_log *P*_classic	7	a	0.44	0.57
HeLa	Consensus_log *P*_ACD	7	a	0.60	0.68
HeLa	ACD_log *P*_galas	7	a	0.74	0.79
HeLa	ACD_log *D*	7	a	0.59	0.68
HeLa	log *P*_chemicalize	7	a	0.81	0.82
HeLa	log *D*_chemicalize	7	a	0.78	0.82
HeLa	CHI_{C18}	7	a	0.79	0.93
HeLa	CHI_{IAM}	7	a	0.87	0.93
HeLa	log *k*_IAM_SPH	7	a	0.90	0.96

Endpoint	Descriptor	*n*	Scenario	*r*	*ρ*
3T3_L1	ACD_log *P*_classic	7	b	0.20	0.36
3T3_L1	Consensus_log *P*_ACD	7	b	0.45	0.50
3T3_L1	ACD_log *P*_galas	7	b	0.69	0.71
3T3_L1	ACD_log *D*	7	b	0.45	0.50
3T3_L1	log *P*_chemicalize	7	b	0.73	0.68
3T3_L1	log *D*_chemicalize	7	b	0.69	0.68
3T3_L1	CHI_{C18}	7	b	0.56	0.70
3T3_L1	CHI_{IAM}	7	b	0.79	0.81
3T3_L1	log *k*_IAM_SPH	7	b	0.73	0.77
MCF7	ACD_log *P*_classic	7	b	0.66	0.56
MCF7	Consensus_log *P*_ACD	7	b	0.66	0.70
MCF7	ACD_log *P*_galas	7	b	0.62	0.71
MCF7	ACD_log *D*	7	b	0.66	0.70
MCF7	log *P*_chemicalize	7	b	0.68	0.70
MCF7	log *D*_chemicalize	7	b	0.66	0.70
MCF7	CHI_{C18}	7	b	0.90	0.93
MCF7	CHI_{IAM}	7	b	0.70	0.78
MCF7	log *k*_IAM_SPH	7	b	0.86	0.89
C6	ACD_log *P*_classic	7	b	0.23	0.54
C6	Consensus_log *P*_ACD	7	b	0.39	0.64
C6	ACD_log *P*_galas	7	b	0.58	0.74
C6	ACD_log *D*	7	b	0.38	0.64
C6	log *P*_chemicalize	7	b	0.66	0.79
C6	log *D*_chemicalize	7	b	0.61	0.79
C6	CHI_{C18}	7	b	0.65	0.89
C6	CHI_{IAM}	7	b	0.75	0.89
C6	log *k*_IAM_SPH	7	b	0.77	0.93
HeLa	ACD_log *P*_classic	7	b	0.44	0.57
HeLa	Consensus_log *P*_ACD	7	b	0.60	0.68
HeLa	ACD_log *P*_galas	7	b	0.74	0.79
HeLa	ACD_log *D*	7	b	0.59	0.68
HeLa	log *P*_chemicalize	7	b	0.81	0.82
HeLa	log *D*_chemicalize	7	b	0.78	0.82
HeLa	CHI_{C18}	7	b	0.79	0.93
HeLa	CHI_{IAM}	7	b	0.87	0.93
HeLa	log *k*_IAM_SPH	7	b	0.90	0.96
3T3_L1	ACD_log *P*_classic	5	c	-0.15	0.20
3T3_L1	Consensus_log *P*_ACD	5	c	0.36	0.60
3T3_L1	ACD_log *P*_galas	5	c	0.87	0.97
3T3_L1	ACD_log *D*	5	c	0.34	0.60
3T3_L1	log *P*_chemicalize	5	c	0.89	0.90
3T3_L1	log *D*_chemicalize	5	c	0.88	0.90
3T3_L1	CHI_{C18}	5	c	0.39	0.60
3T3_L1	CHI_{IAM}	5	c	0.92	0.90
3T3_L1	log *k*_IAM_SPH	5	c	0.73	0.80
MCF7	ACD_log *P*_classic	4	c	0.05	-0.40
MCF7	Consensus_log *P*_ACD	4	c	-0.57	-0.40
MCF7	ACD_log *P*_galas	4	c	-0.62	-0.40
MCF7	ACD_log *D*	4	c	-0.56	-0.40
MCF7	log *P*_chemicalize	4	c	-0.34	-0.40
MCF7	log *D*_chemicalize	4	c	-0.34	-0.40
MCF7	CHI_{C18}	4	c	0.90	0.80
MCF7	CHI_{IAM}	4	c	-0.25	0.00
MCF7	log *k*_IAM_SPH	4	c	0.65	0.60
C6	ACD_log *P*_classic	7	c	0.23	0.54
C6	Consensus_log *P*_ACD	7	c	0.39	0.64
C6	ACD_log *P*_galas	7	c	0.58	0.74
C6	ACD_log *D*	7	c	0.38	0.64
C6	log *P*_chemicalize	7	c	0.66	0.79
C6	log *D*_chemicalize	7	c	0.61	0.79
C6	CHI_{C18}	7	c	0.65	0.89
C6	CHI_{IAM}	7	c	0.75	0.89
C6	log *k*_IAM_SPH	7	c	0.77	0.93

Endpoint	Descriptor	*n*	Scenario	*r*	*ρ*
HeLa	ACD_log *P*_classic	7	c	0.44	0.57
HeLa	Consensus_log *P*_ACD	7	c	0.60	0.68
HeLa	ACD_log *P*_galas	7	c	0.74	0.79
HeLa	ACD_log *D*	7	c	0.59	0.68
HeLa	log *P*_chemicalize	7	c	0.81	0.82
HeLa	log *D*_chemicalize	7	c	0.78	0.82
HeLa	CHI_{C18}	7	c	0.79	0.93
HeLa	CHI_{IAM}	7	c	0.87	0.93
HeLa	log *k*_IAM_SPH	7	c	0.90	0.96

Table S7. Comparison of linear, quadratic, and segmented regression models for the relationship between CHI_{IAM} and vasodilation in the investigated bisphenol analogues

Model	*n*	R^2	R^2_{adj}	AIC	BIC	RSE	MAE
Linear	13	0.044	−0.043	114.3	116.2	24.41	19.69
Quadratic	13	0.620	0.544	107.5	110.3	16.14	12.13
Segmented	13	0.806	0.741	101.8	105.4	12.17	6.62
Linear low-to-moderate CHI_{IAM}	8	0.982	0.978	42.1	42.8	2.96	2.41

RSE: residual standard error; MAE: mean absolute error; AIC: Akaike information criterion; BIC: Bayesian information criterion.

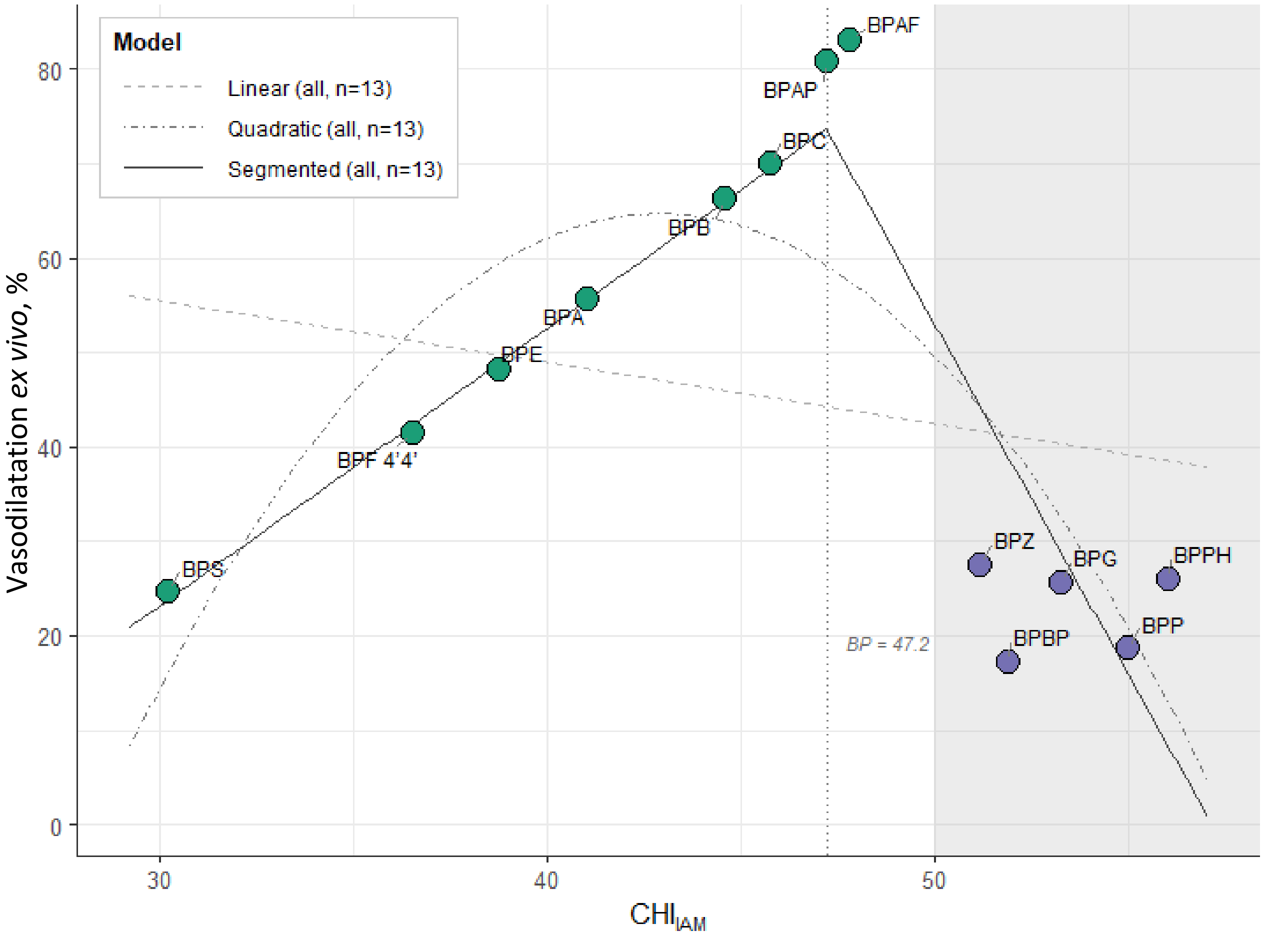

Figure S1. Relationship between CHI_{IAM} and bisphenol-induced vasodilation with fitted regression models: simple linear (grey dashed), quadratic (grey dot-dash), and segmented piecewise regression (grey solid). The vertical dotted line indicates the estimated breakpoint (BP = 47.2 ± 1.2). The shaded area denotes the high-affinity regime (CHI_{IAM} > 50). Compounds are colour-coded by group: green (linear) and purple (deviation)

Table S8. Cook's distance influence diagnostics for individual compounds in the linear regression model of CHI_{IAM} and vasodilation in the investigated bisphenol analogues.

Compound	Group	CHI_{IAM}	Vasodilation, %	Cook's D
BPS	Linear	30.23	24.65	1.0304
BPAF	Linear	47.84	83.14	0.1242
BPAP	Linear	47.21	80.73	0.1031
BPP	Deviation	55.01	18.61	0.1010
BPBP	Deviation	51.91	17.28	0.0776
BPPH	Deviation	56.05	26.01	0.0458
BPC	Linear	45.77	69.96	0.0464
BPG	Deviation	53.28	25.66	0.0371
BPB	Linear	44.60	66.18	0.0324
BPF 4'4'	Linear	36.52	41.49	0.0259
BPZ	Deviation	51.17	27.53	0.0242
BPA	Linear	41.05	55.69	0.0065
BPE	Linear	38.75	48.24	0.0004
